# Systemic Inflammatory Response Syndrome Following High-Dose Intravenous Glutathione-Containing Revitalising Solution in a Patient on Tirzepatide: A Case Report

**DOI:** 10.7759/cureus.84736

**Published:** 2025-05-24

**Authors:** Deepak Sharma, Sarala Daram, Priyanka Sharma

**Affiliations:** 1 Critical Care Medicine, Sandwell and West Birmingham Hospitals National Health Service (NHS) Trust, Birmingham, GBR; 2 Clinical Research, University of Birmingham, Birmingham, GBR

**Keywords:** cosmetic intravenous therapy, endotoxin contamination, glutathione infusion, nutritional deficiency, systemic inflammatory response syndrome, tirzepatide, unregulated supplements

## Abstract

Intravenous glutathione-based products are increasingly used for cosmetic purposes despite lacking regulatory approval in the United Kingdom and robust safety data. We report a case of systemic inflammatory response syndrome (SIRS) following administration of Glutax 75GX DCRP, a high-dose unregulated glutathione infusion, in a patient on tirzepatide with prolonged low nutritional intake. A previously healthy woman presented with shock, hyperpyrexia (>41°C), and collapsed within an hour of receiving the infusion for skin lightening. She had been self-administering tirzepatide for weight loss, leading to significant dietary restriction. Laboratory findings included marked leucocytosis (white cell count (WCC), 26 × 10⁹/L), elevated inflammatory markers (C-reactive protein (CRP), 160 mg/L, procalcitonin 28.8 µg/L), acute liver injury (alanine transaminase (ALT), 311 IU/L), and coagulopathy (prothrombin time (PT), 26.4 s), with no infectious source identified. The patient recovered fully with supportive care. We hypothesise that SIRS resulted from either endotoxin contamination of the unregulated product or a synergistic effect of supraphysiological glutathione in a nutritionally compromised host, as supported by literature documenting similar reactions to unregulated infusions. This case highlights the dangers of cosmetic intravenous therapies administered in non-clinical settings and underscores the need for thorough medication and supplement histories in acute presentations. Clinicians should maintain heightened suspicion for such products in unexplained systemic inflammation and advocate for stricter regulatory oversight to mitigate risks.

## Introduction

The use of intravenous glutathione, vitamins, and related antioxidant cocktails for cosmetic purposes, particularly for skin lightening and anti-ageing purposes, has become increasingly popular worldwide, most likely because of the widespread promotion of their purported benefits on social media. These infusions are often administered in non-medical settings and are not approved by regulatory authorities such as the Food and Drug Administration (FDA) or European Medicines Agency (EMA) [1-3]. Severe adverse reactions, including systemic inflammatory response syndrome (SIRS), have been reported but remain under-recognised. SIRS is a clinical condition characterised by widespread inflammation and physiological derangements, such as fever, tachycardia, tachypnoea, and abnormal white cell count, that can result from infection, trauma, or exposure to toxins [1]. We present a case of SIRS following the administration of Glutax 75GX DCRP, a high-dose glutathione-based product, in a patient on tirzepatide - a dual GIP and GLP-1 receptor agonist used for diabetes and weight management - with significant nutritional compromise [4].

## Case presentation

A woman in her 30s, with no significant past medical history, presented to the emergency department after sudden collapse, vomiting, and loss of consciousness within an hour of receiving an intravenous injection of Glutax 75GX DCRP. She had been self-administering tirzepatide for weight loss, obtained from an online pharmacy, and reported a prolonged period of markedly reduced nutritional intake. She started with a 2.5 mg subcutaneous injection every week three months ago and had titrated up to 7.5 mg weekly at the time of presentation. She had no known drug allergies or significant past medical history. Socially, she was normally fit and well and independent in all activities of daily living.

On arrival, she was found to be in shock with profound hypotension, a systolic blood pressure in the range of 50-60 mmHg. She was tachycardic with a heart rate of 124 beats per minute and hyperpyrexic, with a recorded temperature exceeding 41°C. Her Glasgow Coma Scale (GCS) was 14/15, indicating mild drowsiness but no significant alteration in consciousness. Initial intravenous fluid resuscitation did not produce a haemodynamic response. With sepsis being a differential diagnosis at the onset, she was given broad-spectrum antibiotics, and cultures were taken for blood and urine.

On physical examination, she appeared drowsy and flushed. There was no evidence of urticaria or angioedema, and she exhibited no focal neurological deficits. Unfortunately, after being refractory to ongoing fluid resuscitation, she was admitted to the intensive care unit (ICU) for ongoing treatment with vasopressors. There was no airway or breathing compromise at any time.

Investigations

Laboratory assessment revealed a marked systemic inflammatory response, acute hepatic and renal dysfunction, and coagulopathy, with no evidence of infection, anaphylaxis, or exogenous toxin exposure, apart from the cosmetic infusion additives (Table 1). Imaging studies, including chest radiography and abdominal ultrasound, revealed no acute abnormalities. Microbiological investigations, including blood and urine cultures and screening for blood-borne viruses, were negative. Electrocardiography on admission showed sinus tachycardia at 124 bpm, with a QRS duration of 120 ms and a QTc of 386 ms.

**Table 1:** Initial Laboratory and Investigation findings with plausible interpretation

Test	Result	Reference range		Interpretation
White Cell Count (WCC)	26 × 10⁹/L	4–11 × 10⁹/L		Marked leucocytosis
C-reactive Protein (CRP)	160 mg/L	<5 mg/L		Significantly raised
Procalcitonin	28.8 µg/L	<0.5 µg/L		Considerably elevated
Alanine Transaminase (ALT)	311 IU/L	10–40 IU/L		Significant hepatic injury
Bilirubin	44 µmol/L	<21 µmol/L		Hyperbilirubinemia
Creatinine	110 µmol/L	45–90 µmol/L (female)		Mild acute kidney injury (resolved within 24 hours)
Prothrombin Time (PT)	26.4 s	11–15 s		Coagulopathy
pH (arterial)	7.35	7.35–7.45		Borderline low
Base Deficit	–6.6 mmol/L	–2 to +2 mmol/L		Moderate metabolic acidosis
Lactate	5 mmol/L	<2 mmol/L		Elevated (suggests hypoperfusion)
Anion Gap	15.8 mmol/L	8–16 mmol/L		Mildly elevated
Mast Cell Tryptase	Initial: 4.7 µg/L; 24 hours: 4.1 µg/L	<11.4 µg/L		No evidence of mast cell activation
Urine Toxicology	Lidocaine, caffeine detected		-	Common cosmetic infusion additives

Treatment, Outcome, and Follow-up

The differential diagnoses for this patient’s acute shock and systemic inflammatory response included sepsis, anaphylaxis, and toxin-mediated reactions. Sepsis was effectively ruled out by negative cultures, absence of an infectious source, and rapid clinical improvement. Anaphylaxis was considered unlikely due to the absence of cutaneous or airway features and non-elevated mast cell tryptase. Other non-infectious causes of SIRS, such as acute endocrine emergencies, drug intoxication, or metabolic derangements, were excluded based on history, examination, and targeted laboratory testing. The clinical picture, temporal association with the infusion, and exclusion of other causes supported a diagnosis of toxin- or excipient-induced SIRS.

Upon arrival in the ICU, the patient was started on vasopressor (noradrenaline) support due to a lack of response to aggressive intravenous fluid resuscitation to maintain adequate blood pressure. She received close haemodynamic monitoring, and vasopressors were used to maintain adequate perfusion. Broad-spectrum antibiotics were initially given but stopped after discussions with the microbiology team in the absence of a clear infectious source and prompt resolution of shock. No corticosteroids or antihistamines were administered, as there was no clinical or laboratory evidence of anaphylaxis or allergic reaction. Over the subsequent 24 hours, with ongoing supportive management, the patient’s haemodynamic status gradually stabilised, her temperature normalised, and her laboratory abnormalities began to resolve. By 48 hours, she had made a full clinical recovery and was transferred from the ICU to the ward in a stable condition.

At the two-week follow-up, the patient reported no ongoing symptoms and had returned to her baseline level of health. She was extensively counselled about the risks associated with unregulated intravenous cosmetic therapies and self-administered medications obtained online.

## Discussion

Our case describes a rare but significant systemic reaction in a previously healthy woman following an intravenous injection of Glutax 75GX DCRP, a commercially available compounded solution marketed for skin lightening and revitalisation, containing high-dose glutathione along with other additives such as vitamin C, collagen, and various micronutrients [5]. We propose that the patient’s severe SIRS was triggered by the administration of a high-dose, unregulated intravenous glutathione-based product (Glutax 75GX DCRP) in the context of significant nutritional compromise due to prolonged tirzepatide use. Tirzepatide, a glucagon-like peptide-1 (GLP-1)/gastric inhibitory polypeptide (GIP) receptor agonist, is associated with substantial weight loss and can lead to reduced protein and micronutrient intake over time [4]. We assessed the principles of the Naranjo Adverse Drug Reaction Probability Scale to assess causality, considering factors such as temporal association, exclusion of other causes, and response to withdrawal of the suspected agent. This supported a probable relationship between the infusion and the observed SIRS [6]. Glutathione synthesis is highly dependent on the availability of amino acid substrates, particularly cysteine, glycine, and glutamate, which may be depleted in states of malnutrition [7]. In this scenario, the patient’s antioxidant defences were likely depleted, disrupting redox homeostasis and predisposing her to an exaggerated inflammatory response [5]. Additionally, unregulated compounded products such as Glutax 75GX DCRP are at high risk for endotoxin or other contaminant exposure, which can directly activate innate immune pathways and precipitate SIRS [8,9]. The rapid onset of hypotension, hyperpyrexia, and laboratory evidence of sterile inflammation, in the absence of infection or classic anaphylaxis, supports a non-infectious mechanism. This may involve direct toxic insult, excipient-induced immune activation, or endotoxin-mediated SIRS. This case exemplifies how the combination of nutritional compromise, unregulated infusions, and potential contaminants can lead to life-threatening inflammatory responses.

Severe adverse reactions following intravenous glutathione administration have been described in the literature, although they remain rare. Most reported cases involve the rapid onset of symptoms, such as flushing, nausea, hypotension, and collapse, typically occurring within minutes to hours of infusion. In many instances, no infectious source is identified, and patients recover rapidly with supportive care, further supporting a non-infectious toxin-mediated aetiology [8, 10]. Recent case reports have further underscored the risks associated with unregulated intravenous cosmetic therapies. Multiple published reports and reviews document that intravenous glutathione for cosmetic use can cause severe adverse reactions, including anaphylaxis and life-threatening events. Several published reports and reviews have documented that intravenous glutathione used for cosmetic purposes can lead to severe adverse reactions, including anaphylaxis, with recognition of these rare but serious events by both dermatologists and regulatory authorities [10-12]. These cases align with the findings of Zubair et al. (2016), which reported a case of anaphylactic shock after IV glutathione for skin lightening [13]. These cases collectively underscore a concerning pattern of sterile systemic reactions to unregulated infusions. The absence of infectious sources and consistent resolution with supportive care across cases reinforce the likelihood of toxin- or contaminant-mediated mechanisms rather than classical allergic or infectious aetiologies. These findings emphasise the need for heightened clinical suspicion of such products in cases of unexplained systemic inflammation and advocate for stricter regulatory oversight of cosmetic intravenous therapies.

Regulatory agencies, including the Philippine FDA and US FDA, have issued public warnings against the use of intravenous glutathione for aesthetic purposes, citing the lack of clinical trial data, unknown long-term safety profiles, and risk of contamination in compounded preparations [2,3]. There are currently no approved clinical guidelines that endorse intravenous glutathione for cosmetic use, and major dermatological organisations recommend against such interventions outside of clinical trials. The literature also highlights the potential for nutritional deficiencies to impair glutathione synthesis and antioxidant defences, increasing vulnerability to oxidative and inflammatory stress [7]. Although direct causality between tirzepatide-induced malnutrition and SIRS cannot be proven in a single case, the biological plausibility is supported by mechanistic studies and case reports. Thus, while contamination remains the most likely proximate trigger, the interplay of high-dose glutathione administration and underlying nutritional depletion likely contributed to the severity of the reaction in this patient. This case underscores the importance of thorough history-taking regarding cosmetic infusions and weight-loss agents in patients presenting with unexplained SIRS and highlights the urgent need for greater regulatory oversight and public education regarding unregulated injectable therapies.

## Conclusions

Unregulated IV glutathione products can cause life-threatening SIRS, particularly in patients with nutritional compromise due to weight-loss medications such as tirzepatide. Healthcare providers should remain vigilant and systematically screen for non-prescription cosmetic infusions and weight-loss medications when evaluating patients with unexplained systemic inflammation. Further research is needed to understand the interplay between nutritional status, weight-loss pharmacotherapy, and antioxidant therapies.
